# Droplet evaporation on porous fabric materials

**DOI:** 10.1038/s41598-022-04877-w

**Published:** 2022-01-20

**Authors:** Marta Gonçalves, Jin Young Kim, Yeseul Kim, Najaf Rubab, Narina Jung, Takeshi Asai, Sungchan Hong, Byung Mook Weon

**Affiliations:** 1 Soft Matter Physics Laboratory, School of Advanced Materials Science and Engineering, SKKU Advanced Institute of Nanotechnology (SAINT), Sungkyunkwan University, Suwon, 16419 South Korea; 2 Research Center for Advanced Materials Technology, Sungkyunkwan University, Suwon, 16419 South Korea; 3 Korea Institute for Advanced Study, Seoul, 02455 South Korea; 4 Faculty of Health and Sports Science, University of Tsukuba, Tsukuba, 305 8574 Japan

**Keywords:** Materials science, Physics

## Abstract

Droplet evaporation on porous materials is a complex dynamic that occurs with spontaneous liquid imbibition through pores by capillary action. Here, we explore water dynamics on a porous fabric substrate with in-situ observations of X-ray and optical imaging techniques. We show how spreading and wicking lead to water imbibition through a porous substrate, enhancing the wetted surface area and consequently promoting evaporation. These sequential dynamics offer a framework to understand the alterations in the evaporation due to porosity for the particular case of fabric materials and a clue of how face masks interact with respiratory droplets.

## Introduction

Droplet evaporation and imbibition in porous materials are ubiquitous phenomena in nature that are also relevant in diverse industrial processes, such as textile engineering and device fabrication through ink-jet printing^1–10^. The evaporation of a droplet on a flat solid substrate is a complex fundamental physical phenomenon that has been well studied theoretically, experimentally, and numerically^11–13^. However, unlike these previously mentioned dynamics, a droplet on porous substrates encounters not only spreading at the surface but also imbibition into the media, which would significantly affect droplet evaporation dynamics^14–16^. This complexity introduced by the porous network and the difficulty in visualizing the liquid behavior inside the porous media demonstrates how the study of droplet evaporation on porous materials is a challenging yet very important topic^17,18^.

**Figure 1:**
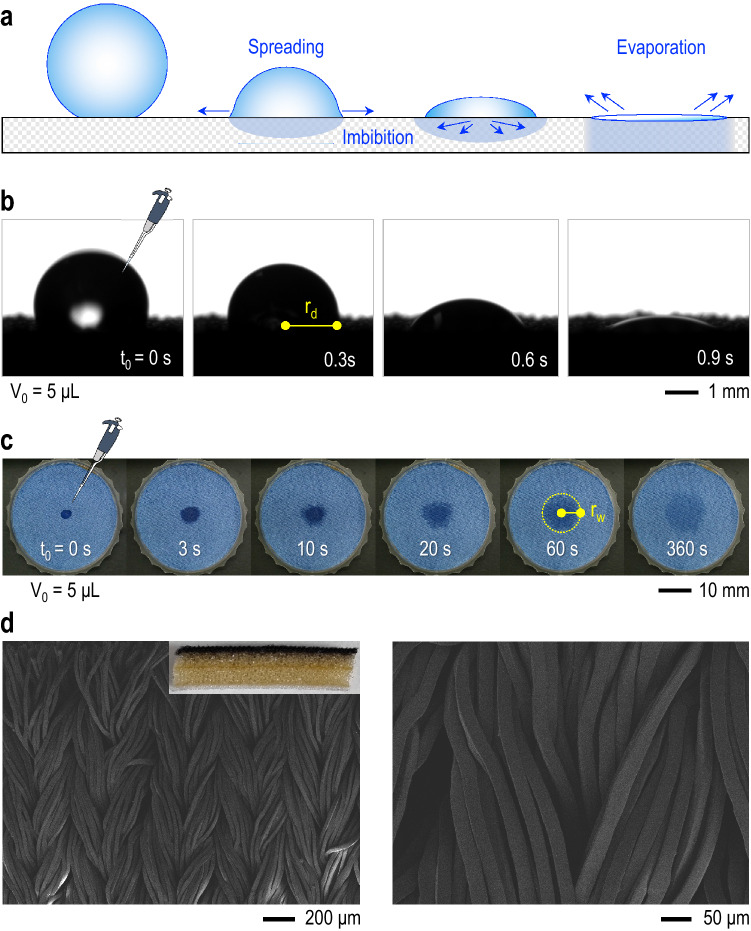
Droplet evaporation on porous media. **(a)** A combination of spreading, imbibition, and evaporation dynamics for a droplet on a porous substrate. **(b)** Side-view observation of a water droplet contact radius ($r_{d}$) and surface area variation on a porous substrate (Acquired with Advance Software, Krüss Science). **(c)** Top-view observation of a radius ($r_{w}$) corresponding to the wetted surface area on a porous substrate. Here, $t_0$ corresponds to the droplet deposition onto the porous substrate. **(d)** SEM images of the porous fabric material surface (Acquired with S-3000H, Hitachi). The inset digital-camera image shows the complete fabric structure.

In porous substrates, the wetting of the surface generates negative capillary pressure, leading to spontaneous imbibition of the liquid into the substrate: this phenomenon is known as *wicking*^19–21^. The wicking flow into porous substrates is dependent on the material properties, impacting the spreading of absorbed liquids on the substrate^22–25^. A general combination of spreading, imbibition, and evaporation dynamics for a liquid droplet on a porous substrate is schematically illustrated in Fig. 1a. For porous media, the spontaneous movement of liquid by capillary pressure was previously described by Lucas^26^ and Washburn^27^ as a linear relationship between the imbibition rate and the square root of time. This relationship is expressed in the Lucas-Washburn equation, $l = (\frac{\sigma b \cos \theta }{2\mu }t)^{1/2}$, where *l* is the length of penetration, $\sigma$ is the surface tension, $\theta$ is the equilibrium contact angle, $\mu$ is the dynamic viscosity, and *b* is the pore radius^28^. The evaporation rate, being proportional to the contact radius of sessile droplets, would inevitably be affected by spreading and imbibition dynamics on a porous substrate due to the variation on the droplet surface area, as shown in Fig. 1b, and enhancement of the wetted surface area, represented in Fig. 1c^29,30^. Regarding droplet evaporation on porous substrates, how spreading and imbibition promote evaporation is a central question.

The surface structure and properties of the most diverse products composed by porous substrates are dependent on their applications, determining different interactions between the liquid and the material. For instance, commercial printing paper with preferential hydrophobic coating controls the liquid imbibition, whereas absorbent paper towels will promote the wicking flow^31^. Additionally, in sportswear industry, moisture control through absorption and evaporation is crucial in managing the external fluids and inner body sweat that inevitably come in contact with the fabric, offering protection and comfort to athletes^32,33^. To achieve breathability and waterproofing, appropriate textiles such as multilayered textiles are being developed^34–36^. Furthermore, the study of droplet evaporation in fabric materials, such as protective fabric masks, can enlighten the interaction of mucoidal viral droplets with complex porous networks, resulting in a better perception on their efficiency and handling^37^. Understanding the combined dynamics of spreading, wicking, and evaporation is therefore indispensable to improve moisture management in textiles^38^.

Here, we aim to understand how spreading and wicking dynamics affect the evaporation dynamics of a sessile droplet on a porous fabric material (Fig. 1d). To achieve this, we experimentally explore evaporation dynamics by measuring the evaporation rates of water droplets on porous and non-porous substrates. Additionally, we observe the progression of water droplet surface area and extended wetted surface area with time due to the porous nature of the sample to determine spreading and wicking dynamics. By performing X-ray imaging and optical imaging, we are able to observe water dynamics in porous substrates. X-ray microscopy and microtomography provide information of the events inside the sample with both two and three dimensional data^39–42^ and optical imaging provides large-scale information. Based on the experimental results, we discuss the physical mechanisms underlying how wicking extends the wetted surface area and consequently promotes evaporation in porous substrates.

## Methods

We examined three kinds of substrates with distinct pore properties for comparison: two porous (a fabric for a ski suit and a commercial printing paper) and a non-porous (flat PDMS, where PDMS = polydimethylsiloxane) substrates. The selected fabric substrate is a five-layered laminated fabric used in ski jumping suits. This material is composed by outer layer, inner layer (two foam layers with an elastic membrane in the middle), and lining fabric, comprising a total thickness of 4.0 mm. The layers connect to each other by hot-melt process, creating in-between adhesive layers. The fabric presents a controlled air permeability of 40 l/m^2^/s. The outer layer and lining layer are a bi-elastic warp-knit fabric, consisting of 81% polyamid gloss and 19% elasthane. Scanning electron microscopy analysis (S-3000H, Hitachi) was performed to provide additional information about the substrate surface. During experiments, the fabric substrates were placed in petri dishes to prevent the vapor diffusion in horizontal direction from the evaporation of absorbed water into porous substrate. The selected commercial printing paper (80 gsm premium multi-purpose, Double A ©) has a coated surface to restrict the amount of liquid absorbed into the paper, leading the droplet to preferentially stay on the surface. The flat PDMS is a solid, non-porous substrate with a similar initial contact angle, chosen as a control sample where imbibition is not present.

For spreading and imbibition evaluations, we obtained both top-view and side-view images of the droplet (5 $$\upmu$$l) deposited on different substrates (fabric, commercial printing paper, and flat PDMS substrates). For the fabric sample (marked by ‘porous media’), three droplet volumes (5, 7, and 10 $$\upmu$$l) were tested to access the scale effect. The side-view optical images of the droplet were acquired by a droplet shape analyzer (DSA25, Krüss) equipped with a back light and a charge-coupled device (CCD) camera. This allowed to record the droplet contact radius ($$r_d)$$ and contact angle ($$\theta$$) of the droplet during spreading in real time, automatically acquired each second. The top-view optical images were taken with a digital camera (Samsung EV-NX300MAST, 18-55 mm lens) mounted on a tripod facing the droplet vertically, allowing to detect the radius of the wetted surface area, $$r_w$$. The acquired images were further analyzed with ImageJ software (Maryland, USA). The acquired data were processed by using the Origin 2021 (OriginLab Corp., Massachusetts).

For evaporation experiments, the water droplet mass was automatically measured every second during evaporation by using an electronic mass balance (EX224G, Ohaus) with 0.1 mg readability. The mass change with time was measured until complete evaporation of the water droplet, achieved when mass value reached 0 mg. The initial water droplets were set to be 5$\sim$10 $\upmu$l of pure deionized water (DI water system, ELGA) and were carefully delivered with a micro pipette onto the surface of the substrates, placed inside the mass balance. All experiments were conducted at $24\pm 2 \,^\circ$C room temperature and $45\pm 5$% relative humidity.

**Figure 2:**
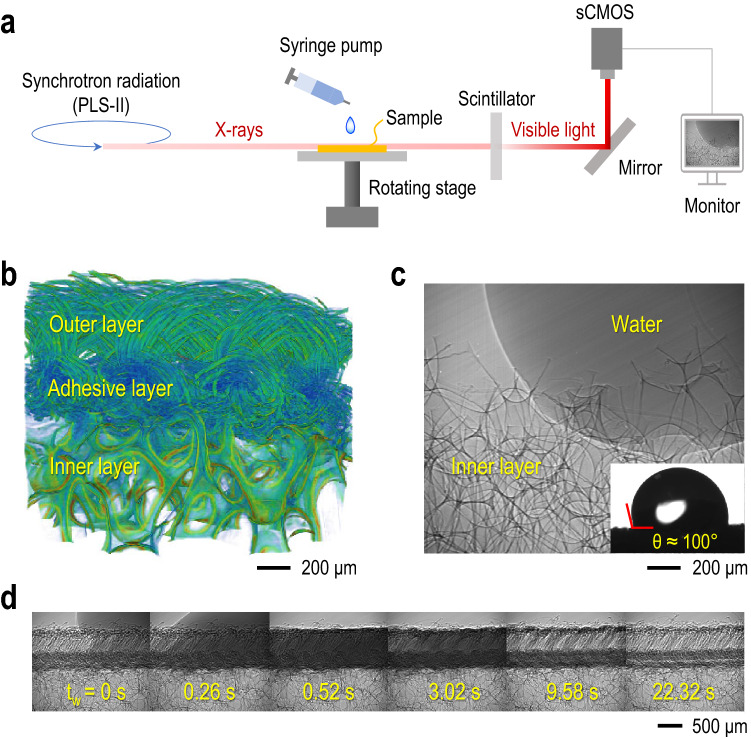
X-ray imaging for 2D microscopic and 3D microtomographic observations of a porous fabric and water dynamics. **(a)** Schematic illustration of the image acquisition process from X-ray microscopy technique (see details in “Methods”). **(b)** 3D reconstruction image of fabric with multiple (outer, adhesive, and inner) layers taken by X-ray microtomography (Generated with Amira software (version 2019.3)). **(c)** Little water imbibition to the inner layer taken by X-ray microscopy (Acquired with Micro-Manager software (version 1.4, https://micro-manager.org)). The inset side-view image represents the high intrinsic contact angle on the inner layer ($\theta \approx 100^{\circ }$). **(d)** Real-time observation of water droplet (1 $\upmu$l) imbibition and evaporation taken by X-ray microscopy, showing that spreading and imbibition are confined in the outer layer (Movie S1). Here, $t_w$ corresponds to the beginning of wicking phenomenon (Acquired with Micro-Manager software (version 1.4, https://micro-manager.org)).

X-ray imaging is a powerful tool in soft matter and fluid dynamics, especially regarding porous media study, where conventional light imaging techniques cannot overcome practical difficulties such as light scattering and low resolution. High-resolution and high-penetration X-ray imaging technique allows in-situ observations of water dynamics inside porous substrates as well as detailed characterizations of the pore network morphology with two-dimensional (2D) microscopic and three-dimensional (3D) microtomographic analyses. X-ray imaging experiments were carried out at the Pohang Light Source II (PLS-II) using the 6C Bio Medical Imaging (BMI) beamline. The beam was characterized by monochromatic synchrotron X-rays with 15 keV energy. Following the sample penetration, X-rays were converted into visible lights by the scintillator (LuAG:Ce 50 $\upmu$m) and collected by the Scientific Complementary Metal-Oxide-Semiconductor (sCMOS) camera (Andor Zyla) with magnification of 10x. The X-ray microscopy image acquisition process is illustrated in Fig. 2a. The effective pixel size was approximately 0.65 $\upmu$m, with a field of view of 1.70$\times$1.40 mm$^{2}$. Using a glass syringe pump, a water droplet was gently placed on a substrate, with volumes ranging from 0.2 to 2.0 $\upmu$l. For morphological characterization, we performed the 3D image reconstruction by applying X-ray microtomography. The 2D projection images taken with X-ray microscopy were recorded from every angle of the sample when rotating from 0 to 180 degrees and the obtained slices were stacked with a program (OCTOPUS, Belgium) to form a 3D image of the sample. The image processing, analysis, and visualization were obtained by using a commercial software Amira (FEI, Hillsboro, Oregon).

## Results and discussion

### X-ray imaging and microtomography of porous fabric materials

From X-ray 2D microscopy and 3D microtomography techniques, clear observations of the water dynamics in a porous fabric substrate are demonstrated in Fig. 2, consistent with the optical observations in Fig. 1b. The ski jumping suit fabric has multiple layers consisting of outer and inner layers connected by a transition adhesive layer (Fig. 2b). The outer layer is characterized by a warp-knit structure (Fig. 1d), being in contact with the exterior. The inner layer, in-between outer and bottom layers, is a foam material characterized by a large inter-yarn pore network, mediating the air permeability. To further investigate the role of multilayered substrates in functional sportswear fabric materials, a water droplet was directly placed onto the surface of the inner layer after detaching the above outer layer. Thanks to direct observations with X-ray microscopy, we find that the absorbed water does not penetrate into the inner layer immediately (Fig. 2c). This absence of water imbibition in the porous inner layer is due to the high intrinsic equilibrium contact angle ($\theta \approx$ 100$^{\circ }$, Fig. 2c inset)^43,44^. This observation follows the principle from the Lucas-Washburn equation, in which the existence of liquid imbibition on a porous substrate depends on the wettability function ($\sigma \cos \theta$). Thus, the inner layer hydrophobicity (also extended into the extremity of the transition layer) confines the water penetration only into the outer layer and partially in the upper part of the adhesive layer that retains similar properties. Here, the absorbed water mainly spreads horizontally along with the outer layer thickness, preventing water from reaching the user of a sportswear product constituted by this material. X-ray microscopy is again a robust tool to verify the water fate that mostly remains on the outer layer, where spreading and wicking dynamics occur. As demonstrated in Fig. 2d, the water droplet is immediately absorbed and spreads horizontally across the outer layer, pointed by the dark gray coloration, enhancing the wetted surface area. Therefore, X-ray enabled the characterization of the imbibition, where the wetted volume is confined between open-air and the inner layer, leading to a saturated imbibition state in the outer layer (Fig. 2d), underneath the wetted surface area as observed in Fig. 1c. Eventually, the water imbibition accelerates the water evaporation in the outer porous layer, denoted by the color change from dark gray to light gray.

### Droplet spreading and imbibition dynamics on porous fabric materials

**Figure 3:**
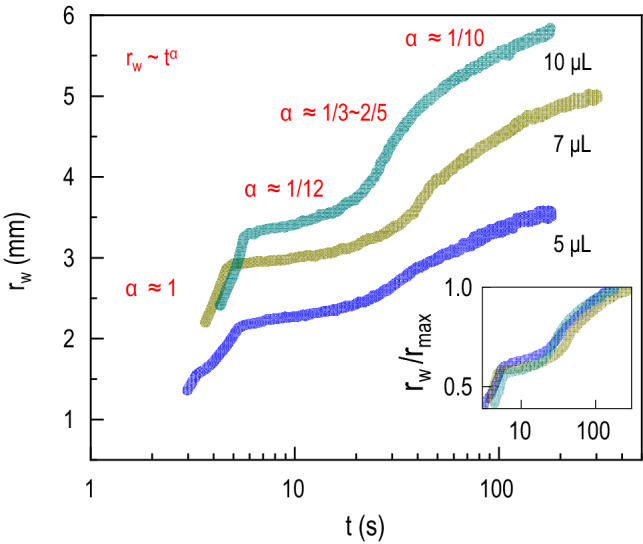
Wetted area radius evolution at early times. Wetted area radius $r_w$ change with time during $0< t < 200s$ for the fabric substrate, where different initial volumes are compared. The exponent $\alpha$ in the power-law scaling $r_w \sim t^{\alpha }$ are represented for each slope. The inset represents the nondimensionalized data ($r_w/r_{max}$ (maximum $r_w$)) to compare the power-law scaling between different droplet volumes.

To relate the spreading of the droplet and imbibition through the porous substrates to the enhancement of the absorbed liquid fronts on the substrates, we measure the contact angle $\theta$, droplet radius $r_d$ and wetted radius $r_w$ change with time from the side view and top view of the droplets. In Fig. 3, the log-log plot of the wetted radius $r_w$ (see Fig. 1c) where $0< t < 200$ s is illustrated for three different initial volumes (5, 7, and 10 $\upmu$l) for the fabric substrate. Interestingly, four distinct slopes are found in the curves, indicating four distinct dynamics of spreading and wicking through the fabric. We quantitatively analyze these behaviors by applying a power-law scaling $r_w \sim t^{\alpha }$, where the exponents $\alpha$ are estimated and presented in Fig. 3. The estimated values of the slopes continuously change with time as $\alpha \approx 1$ at the first stage, $\alpha \approx 1/12$ at the second stage, $1/3 \le \alpha \le 2/5$ at the third stage, and $\alpha \approx 1/10$ at the forth stage.

**Figure 4:**
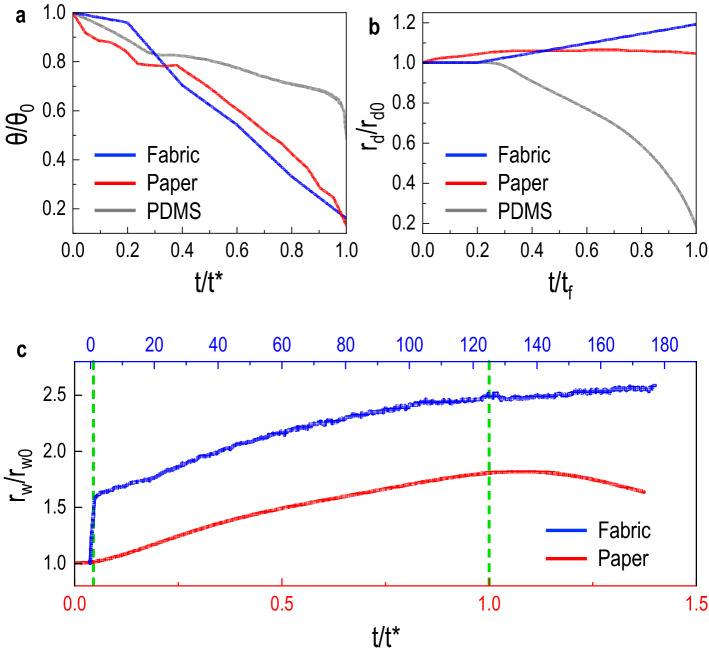
Spreading and imbibition dynamics. **(a)** Contact angle $\theta$ decay for the different substrates. The contact angle $\theta$ was normalized with the respective initial value $\theta _0$ for each substrate and the time *t* was normalized with the characteristic time constant $t^*$ corresponding to the moment of $\theta = 0^{\circ }$. For the fabric and paper substrates, $t^*$ specifically refers to when $\theta \approx 0^{\circ }$. **(b)** Droplet contact radius $r_d$ with time *t* for the different substrates. This graph represents the normalized values to the initial droplet radius $r_{d0}$ and the final time $t_f$. **(c)** Wetted area radius $r_w$ for the porous samples (fabric and paper). The $r_w$ values are normalized to its initial values $r_{w0}$ and the time is normalized to the characteristic time constant $t^*$. The green lines represent the moment when $\theta \approx 0^{\circ }$ for each substrate.

Before we discuss the distinct values of $\alpha$ that depend on the spreading and wicking dynamics of water on the porous substrate, we compare the droplet geometry evolution with time for three substrates: the outer layer of the fabric substrate, commercial printing paper, and flat PDMS. As previously described in Fig. 1, due to spreading and imbibition, the droplet fast transitions from a spherical cap shape to a wetted patch is $\sim 1$ s for the fabric substrate, as the contact angle decays to zero. After that, the wetted radius will increase up until it reaches a constant area exposed to evaporation, later becoming undetected. In contrast, for the paper substrate with hydrophobic properties, the droplet spreading and imbibition occur in a much longer duration (Movie S2 and Movie S3), remaining with a spherical cap shape for the most time and getting completely absorbed into the substrate at $\sim 1300$ s. There is no imbibition into the substrate in the PDMS substrate, and the droplet geometry is only governed by evaporation. These observations are represented quantitatively in Fig. 4. Initially, we compare the contact angle decay by normalizing the contact angle $\theta$ with the respective initial value $\theta _0$ for each substrate and normalizing the time *t* with the characteristic time constant $t^*$, in seconds, corresponding to the moment of last acquired value of $\theta$, which in the case of the porous samples (fabric and paper) its when $\theta \approx 0^{\circ }$. In Fig. 4a, similarly to the described observations, the droplets on fabric and paper substrates ultimately decay to $\theta \approx 0^{\circ }$^45^. However, the droplet on the PDMS passes from a constant contact radius (CCR) mode, where the contact angle decreases initially, to an almost unaltered contact angle stage, now evaporating in constant contact angle (CCA) mode. Lastly, mixed-mode evaporation results in the change in both parameters^46^.

Simultaneously, the droplet contact radius $r_d$ was also acquired and the normalized values to the $r_{d0}$ and final time $t_f$ are represented in Fig. 4b. From this data, we can depict that the contact radius of the droplet on the fabric substrate slightly increases in the very fast-spreading stage. Then due to imbibition, the droplet disappears under the same contact radius, not being possible the observation by the side view afterward^28^. Similar behavior is present for the paper substrate, where the contact radius does not decrease over time, and after imbibition is completed, the droplet is no longer observed. As described for the PDMS, the acquired $r_d$ is initially constant and then transitions to a CCA mode. From this data, for the substrates where imbibition is present, we can identify when the droplet is no longer visible from the side visualizations when $\theta \approx 0^{\circ }$. From that moment forward, coupled side view and top view visualizations are required to track the progression of the wetted area radius $r_w$, until the wetted patch is faded and not quantifiable anymore, as seen in Fig. 1c.

The measured wetted area radius $r_w$ for fabric and paper substrates were normalized to its initial value $r_{w0}$ and the time was normalized to the characteristic time constant $t^*$ from Fig. 4a, represented in Fig. 4c. The characteristic time constant $t^*$ for fabric and paper are 1.03 s and 1260 s, respectively. This data highlights that the complete imbibition of droplets total volume on the fabric occurs at the very beginning, leading afterwards to a much increased wetted area patch when compared to the paper substrate. In the case of paper, the wetted radius slowly increases over time while the water source droplet remains at the surface and is subjected to evaporation. Once the droplet is completely absorbed into the paper, a turning point can be depicted by the decreasing $r_w$, presumably by the dominant evaporation effect at the surface of the wetted area. Chatterjee *et* *al*^45^ reported that for the substrate which displays a larger wetted area, the lifetime of the patch is much smaller. Similar observations were made, where the paper wetted area remains much longer ($t \approx 1500$ s) than the fabric wetted patch ($t \approx 400$ s), presumably explained by the evaporation enhancement due to a larger wetted area. This analysis enlightens that the expansion of wetted radius $r_w$ is present for the porous substrates and that early time spreading and imbibition dynamics greatly dictate the magnitude and evolution of the wetted area subjected to evaporation. By paring up the measured parameters, the surface area *S* subjected to evaporation can be compared for the three substrates in the study.

**Figure 5:**
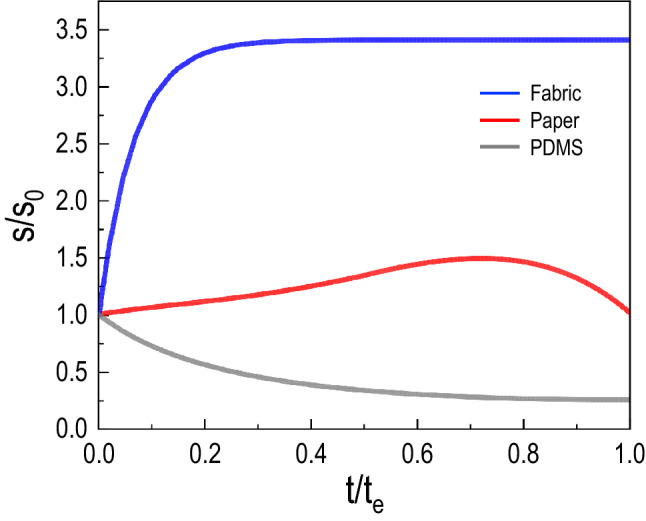
Surface area evolution subjected to evaporation. The temporal surface area *S* contributes to evaporation flux changes for the different substrates. The surface area values are normalized to the initial values $S_0$, and the time *t* is normalized to the experimental final acquisition time $t_e$.

On the non-porous substrates, the droplet surface area (*S*) contributing to evaporation flux is the same as the surface area of the spherical-cap droplet: $S = \pi (r_d^2 + h_d^2)$, where $r_d$ is the contact radius of the droplet and $h_d$ is the maximum height of the droplet. On the porous substrates, *S* becomes larger than the initial contact area with time due to the wetted area enhancement (e.g., see the panels where $t >1$ s in Fig. 2c): therefore, the wetted radius is $r_w > r_d$. In this case, *S* is the sum of the surface area of the spherical cap and the annulus area extended outside of the spherical cap: $S = \pi (r_d^2 + h_d^2) + \pi (r_w^2-r_d^2)$. For the fabric, $S \approx \pi r_w^2$ as $h_d \approx 0$ by the initial rapid imbibition (Fig. 1b). The obtained surface areas for each substrate are plotted in Fig. 5, where *S* was normalized to its initial value $S_0$ and time was normalized to the end time of data acquisition $t_e$ from the experiments. Particularly, as the spreading and imbibition spontaneously occur at the very early times, the wetted surface area in the fabric rapidly increases and becomes saturated at $t > 200$ s. Interestingly, for the paper substrate, the imbibition is hindered by the substrate hydrophobicity, leading to the slow growth of the wetted surface area compared to the porous fabric substrate. The wetted surface area reaches a maximum value at the late stage after the droplet is completely absorbed, leading to a dominant evaporation rate over the spreading rate, decreasing afterward. For the flat PDMS, the droplet surface area monotonically decreases for the entire time because the evaporation monotonically occurs without spreading accompanied by imbibition. Consequently, the enhancement of wetted surface area strongly depends on the wettability of the substrate that permeates the water spreading and imbibition.

### Droplet evaporation dynamics on porous fabric materials

**Figure 6:**
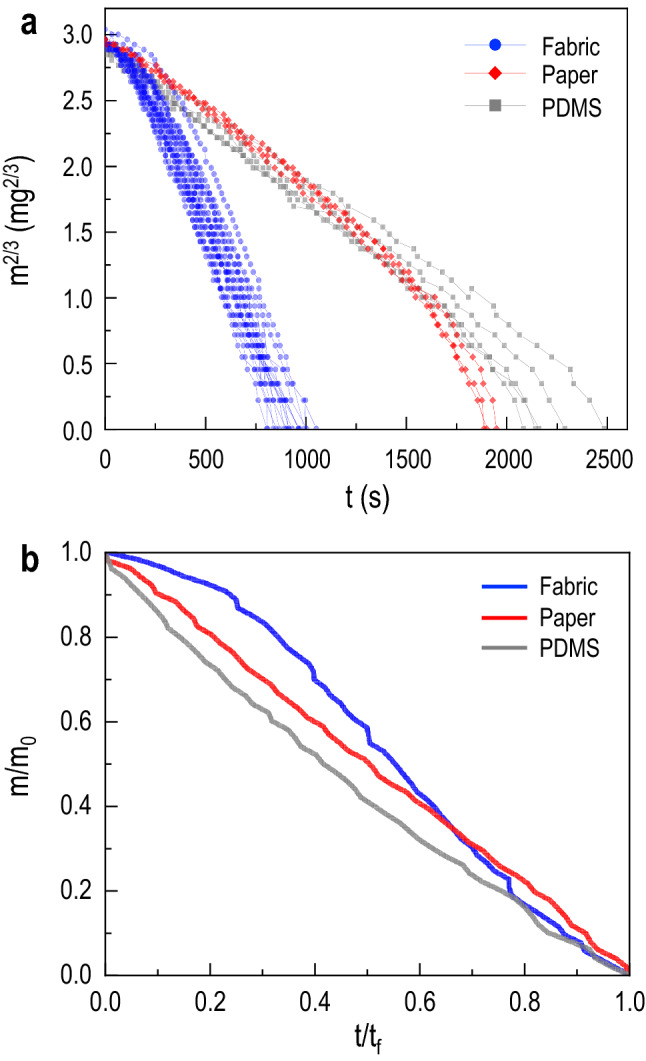
Droplet evaporation dynamics for porous and non-porous substrates. **(a)** The linearity in water mass $m^{2/3}$ versus time *t* indicates the diffusion-limited evaporation of water vapor, which governs most of the evaporation times. A deviation from this linearity indicates how substrate properties affect the evaporation dynamics of a sessile droplet. The fabric substrate displays the highest evaporation rate. **(b)** Distinct evaporation behavior is depicted by comparing the different substrates. Here, the mass *m* is normalized to its initial value $m_0$ and the time *t* is normalized to the final evaporation time $t_f$.

We now analyze the evaporation dynamics of the three different substrate to verify the influence of the distinct exposed wetted areas according to the substrate wettability, as discussed in Fig. 5. The water droplets initial volume are 5 $\upmu$l for the three substrates. Their masses *m* are measured with time and the changes of $m^{2/3}$ during $0<t<2500$ s are plotted in Fig. 6a. The measured mass was converted to a power-law scaling, $m^{2/3}$ versus *t*. Here, the data linearity indicates the diffusion-limited evaporation of water and a deviation from this linearity can be a measure for how the substrate properties affect the evaporation dynamics of a sessile droplet^12^. We estimate their rates as $m_0/t_f$ from the slopes of $m^{2/3}$ versus *t* in Fig. 6a, where $m_0$ is the initial mass of liquid and $t_f$ is the final evaporation time. As a result, the evaporation rates are the largest (16.4 $\upmu$g/s) for the fabric substrate compared with the other substrates (7.8 $\upmu$g/s for the paper and 6.4 $\upmu$g/s the flat PDMS)^30^. For most of the evaporation process ($200< t < 1500$ s), $m^{2/3}$ linearly decrease with *t*, indicating that the evaporation rate *dm*/*dt* is proportional to $m^{1/3}$ regardless of the substrates. As discussed in the previous section, depending on the substrate porosity, the early imbibition dynamics will lead to the modified evaporation dynamics.

To understand these distinct evaporation rates, we compare the different substrates by normalizing the mass to its initial value and the time normalized to the final evaporation time $t_f$ in Fig. 6b. Although *m* monotonically reduces with time for all cases, for fabric, the initial evaporation rates become accelerated in the early stage as depicted by the concave down curve, corresponding to the fast imbibition stage in Fig. 1c. Afterward, the wetted radius becomes constant, slowing down the evaporation. In contrast, the evaporation rate for the flat PDMS linearly decreases throughout the total time, displaying a slight convex curve for slower evaporation. Meanwhile, for the paper substrate, the water imbibition is hindered by a hydrophobic coating (Movie S2). Therefore, the evaporation rates of the paper are slower than the fabric substrate and comparable to the PDMS substrate since both paper and PDMS do not display a rapid early time enhancement of the wetted radius, seen in Figs. 4 and  5. Interestingly, the evaporation rates of the paper slightly increase at the late stage ($t > 1500$ s) when water is eventually absorbed into the paper, seen in Fig. 6a and validated by the data in Fig. 4. By coupling this analysis with the discussion in the previous section, the results indicate that the substrate wettability, leading to the expansion of a wetted area, plays a crucial role in determining the evaporation dynamics.

### Physics of droplet dynamics on porous fabric materials

**Figure 7:**
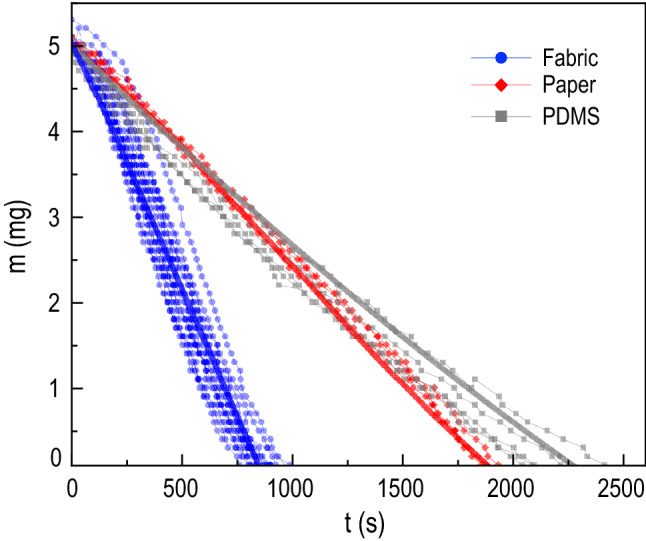
Evaporation dynamics predicted from the enhanced wetted area radius. The solid thick lines exhibit the estimated evaporation dynamics, and the thin lines with dots represent the experimental data for comparison.

As previously described for the porous samples, the spreading and imbibition lead to the generation of a wetted patch with a measured $r_w$ changing with time. This increment of the surface area subjected to evaporation is a characteristic of the porous samples (fabric and paper), as discussed in the previous chapter. To investigate how the experimentally obtained evaporation rates are determined by the expansion of the wetted area contributing to evaporation flux, we describe the evaporation rates $-dm/dt$ by combining the spherical-cap model where ($0^{\circ }<\theta <90^{\circ }$)^47^

1 $$\begin{aligned} -dm/dt=\pi r_d D (1 - H)c_{v} (0.27\theta ^2 + 1.30) \end{aligned}$$

and the free surface where $\theta$ is close to zero, accounting only for the annulus area extended outside of the spherical cap^47^

2 $$\begin{aligned} -dm/dt= 4 \beta D (1 - H)c_{v}(r_w - r_d) \end{aligned}$$

where $r_d$ is the droplet radius, $r_w$ is the wetted radius, *D* is the diffusivity of the water vapor, *H* is the relative humidity, $c_{v}$ the saturated water vapor concentration, and $\theta$ is the contact angle^47,48^. Here, we add $\beta$ as a practical calibration factor for the fabric to get a better fit for the slope. It turns out that $\beta = 1.08$ indicates that the evaporation rate from the free fabric surface is slightly larger than Eq. (2) predicted. The mass change with time for PDMS was predicted by Eq. (1) and for the porous substrates (fabric and paper), Eqs. (1) and (2) were coupled. The expected *m*(*t*) is presented in Fig. 7 by the bold lines. The mass changes determined by the expansion of the wetted area agree well with the experimental data. This agreement indicates that the enhancement of the evaporation rates of the fabric substrate, both experimentally and theoretically represented in Fig. 7, can be explained by the greater expansion of the wetted area compared with paper and PDMS substrates as previously discussed in Fig. 5.

**Figure 8:**
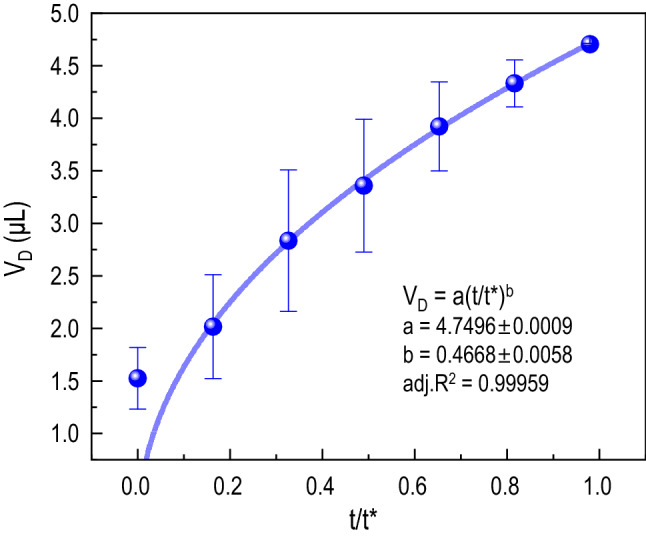
Drained volume dynamics for the fabric substrate. The drained volume $V_D$ with time *t* (normalized to the characteristic time constant $t^*$) is examined with the power law $V_{D} =a(t/t^{*})^{b}$. The fitting result represents a relationship of $V_D \sim t^{1/2}$.

Upon the contact of a droplet with porous substrates, imbibition will simultaneously occur by moving a liquid into the substrate. We compute the drained volume $V_D$ by $V_D(t)=V_0-V(t)$, where $V_0$ is the droplet initial volume and *V*(*t*) is the droplet volume during its life time before complete imbibition, given by the volume of the spherical cap, $V(t)=\frac{\pi r_{d}^3}{12}\left( \frac{2-3\cos \theta +\cos ^3\theta }{\sin ^3\theta }\right)$. In Fig. 8, the evolution of $V_D$ for the fabric sample is plotted, where time *t* is normalized by the characteristic time $t^*$, which was previously obtained from Fig. 4. The power fitting of the plotted data showed that $V_D \sim t^{1/2}$. Chatterjee *et* *al*^45^ previously demonstrated that the drained volume $V_D$ evolves with time as $\sim t^{1/2}$, following the premise from Washburn’s law when evaporation is not significant during the imbibition and the substrate does not have intrinsic hydrophobic properties^43,49,50^. Therefore our result corroborates that in the initial moments, the droplet volume loss is dominated by spreading and imbibition. The same scaling law of $V_D$ does not apply to paper or PDMS substrates, where evaporation plays a much crucial role in the droplet volume loss.

Lastly, we explain the spreading and imbibition dynamics of liquid (5, 7, and 10 $\upmu$l) on the porous media by taking a close look at the exponent $\alpha$ of time for the wetted radius from the power law $r_w \sim t^{\alpha }$ (Fig. 3). We have four characteristic stages in time depicted by the four different $\alpha$ values: (i) when $t < 6$ s where $\alpha = 1$, (ii) when $6< t < 20$ s where $\alpha = 1/12$, (iii) when $20< t < 40s$ where $1/3\le \alpha \le {2/5}$, and (iv) when $t > 40$ s where $\alpha ={1/10}$. As previously shown in Fig. 1b, the imbibition dynamic here described occur in a time scale of three orders of magnitude faster than the evaporation dynamics displayed in Fig. 6a. Additionally, the spreading of the wetted patch until saturation that follows imbibition is also much faster than evaporation dynamics. Therefore, the evaporation effect is neglected during the four characteristic stages explored here. It mainly occurs after these stages are complete.

(i) At the first stage ($t < 6$ s), $\alpha = 1$ depicts a steep slope for the very early stage of imbibition, during which the droplet is completely absorbed into the fabric and forms the very initial state of the wetted area patch. This fast suction of water into the fabric appears to be a unique property of a ski suit we used so that it induces an immediate removal of water from the surface of the material.
(ii) At the second stage ($6< t < 20$ s), a slow down of the lateral spreading beyond that formed in state (i) is followed, giving $\alpha = 1/12$. This companion stage to (i) occurs while water primarily occupies the outer layer’s depth.
(iii) At the third stage ($20< t < 40$ s), $1/3\le \alpha \le {2/5}$ shows that the wetted radius increment becomes accelerated, depicting that water is now mainly flowing radially, enhancing the spreading of the wetted patch. The radial evolution of the wetting front, where radius change with time as $r_w \sim t^{1/3}$ was previously reported by Xiao et al.^51^ for a three-dimensional radial geometry. Additionally, Xiao et al.^52^ also described a radius change with a time of approximately $r_w \sim t^{2/5}$ for two-dimensional radial imbibition. These observations agree with the behavior described here and indicate the fabric substrate complexity that influences the wetted radius enhancement dynamics.
(iv) In the final stage, the expansion rate of $r_w$ decays compared to the stage (iii) and $\alpha ={1/10}$ because of the finite volume of water. The wetted area is now approaching a saturation point.

## Conclusion

To conclude, we have studied the evaporation of water droplets on porous and non-porous substrates and demonstrated that the evaporation rates can be affected by the water imbibition and increased wetted area depending on the substrates properties. It is shown that the evaporation rate is the largest on the fabric material because the spontaneous imbibition into the porous media accelerates the expansion of the wetted surface area under evaporation. Additionally, the early spreading and imbibition dynamics into the fabric material are described as well as the different stages of the wetted radius enhancement. This work would offer a framework to understand the correlated dynamics among the evaporation, the imbibition, and the wetted area enhancement in drying droplets on porous fabric substrates. Furthermore, the study of porous surfaces is becoming a topic of great importance since understanding how face masks interact with respiratory droplets is crucial for improving preventive measures to control the transmission of respiratory infectious diseases^53^. Importantly, the usage of X-ray computed tomography for the study of porous materials, such as face masks, is a key approach for a detailed analysis and morphological characterization^54^. In summary, our study provides valuable evidence and theory that can be essential in the study of general porous surfaces, including face masks.

## Supplementary Information

Supplementary Video S1.

Supplementary Video S2.

Supplementary Video S3.

Supplementary Information.
