# Supplementary material for: Droplet evaporation on porous fabric materials
Source: Sci Rep. 2022 Jan 20;12:1087. doi: 10.1038/s41598-022-04877-w (PMC8776847; doi:10.1038/s41598-022-04877-w)
Supplement: Supplementary file 4 — Supplementary Information. [file 41598_2022_4877_MOESM4_ESM.pdf]

Scientific Reports

Supplementary Information

Droplet evaporation on porous fabric materials

Marta Gonçalves, Jin Young Kim, Yeseul Kim, Najaf Rubab, Narina Jung, Takeshi Asai, Sungchan Hong*, and Byung Mook Weon*

Brief explanation for supplementary movies:

Name	Snapshot	Comments
Movie S1	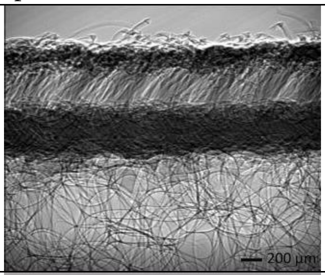	X-ray microscopy video for Fig. 2C – water droplet absorption and evaporation on the porous fabric material.
Movie S2	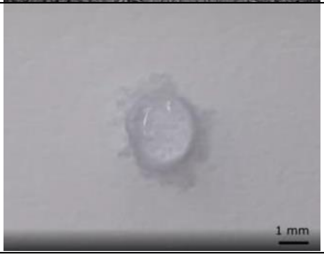	Water droplet behavior on commercial printing paper, top view.
Movie S3	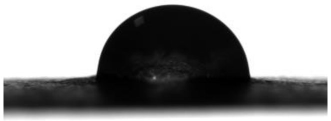	Water droplet behavior on commercial printing paper, side view.

*E-mail: bmweon@skku.edu; sr7931@hotmail.com
